# A multiplex assay based on capillary electrophoresis to detect *Mycobacterium tuberculosis* complex: development and clinical validation

**DOI:** 10.1007/s00253-025-13701-0

**Published:** 2026-02-02

**Authors:** Yaocheng Wang, Zhen Li, Li Lai, Yiping Liu, Li Li, Yi Huang

**Affiliations:** 1 Shengli Clinical Medical College of Fujian Medical University, Fujian Medical University, Fuzhou, China; 2 Central Laboratory, Fujian Provincial Hospital, Fuzhou University Affiliated Provincial Hospital, Fuzhou, China; 3 Clinical Laboratory, Fujian Provincial Hospital, Fuzhou University Affiliated Provincial Hospital, Fuzhou, China; 4 Fujian Provincial Key Laboratory of Cardiovascular Disease, Fuzhou, China; 5 Tuberculosis Laboratory, International Medical Examination Center, Fujian Provincial Hospital, Fuzhou University Affiliated Provincial Hospital, Fuzhou, China; 6 Department of Health Management, Fujian Provincial Hospital, Fuzhou University Affiliated Provincial Hospital, Fuzhou, China; 7 Center for Experimental Research in Clinical Medicine, Fujian Provincial Hospital, Fuzhou University Affiliated Provincial Hospital, Fuzhou, China

**Keywords:** Multiplex assay, *Mycobacterium tuberculosis* complex, Capillary electrophoresis, *IS6110*, *rpoB*, *HSP65*

## Abstract

**Abstract:**

This study presents a novel multiplex assay based on capillary electrophoresis (CE) for the simultaneous detection of three *Mycobacterium tuberculosis* complex (MTBC) genes: *IS6110*, *rpoB*, and *HSP65*. Unlike conventional molecular diagnostic methods that target only a single gene, which may lead to misdiagnosis or missed diagnosis, this CE-based multiplex approach provides comprehensive detection to reduce diagnostic errors. Specificity testing with 76 microorganisms representing common respiratory pathogens confirmed 100% analytical specificity with no cross-reactivity, while sensitivity analysis demonstrated detection limits ranging from 10 to 20 copies/mL for all three target genes. In a prospective clinical validation study of 1067 patients suspected of pulmonary tuberculosis, the multiplex assay showed 77.4% sensitivity (CI 74.9%–79.9%), 99.6% specificity (CI 99.2%–100%), 96.0% positive predictive value (CI 94.8%–97.2%), and 97.1% negative predictive value (CI 96.1%–98.1%). Notably, the study identified 6 MTBC strains (4.8% of TB patients) with *IS6110* deletions through whole-genome sequencing, which would result in false-negative results for any commercial PCR kits targeting *IS6110*. This integrated multiplex approach enhances diagnostic accuracy by simultaneously targeting multiple genes; then it offers the potential to reduce misdiagnosis and missed diagnosis of tuberculosis. In summary, the multiplex assay provides a more comprehensive alternative to current single-target molecular methods for MTBC detection.

**Key points:**

• *The multiplex assay provides one-run results for IS6110, rpoB, and HSP65.*

• *The multiplex assay is a more comprehensive method to detect MTBC.*

• *This approach can reduce misdiagnosis and missed diagnosis of TB.*

**Supplementary Information:**

The online version contains supplementary material available at 10.1007/s00253-025-13701-0.

## Introduction

Tuberculosis (TB), which is caused by *Mycobacterium tuberculosis* complex (MTBC) infection, is still a global health epidemic, showing significant morbidity and mortality (WHO 2025). Indirect evidence suggests that one in four people is infected with MTBC worldwide, and 5%–10% of those develop TB (Houben et al. 2016). According to the World Health Organization (WHO), an alarming 10.7 million people were diagnosed with TB in 2024, and the number of TB-related deaths was about 1.23 million (WHO 2025). This highlights the need to take urgent action for TB prevention. Rapid identification of MTBC is of paramount importance towards early diagnosis of TB, leading to effective infection control.

For the advantages over conventional diagnostic methods like mycobacterial culture, which is time-consuming and acid-fast bacilli (AFB) smear with low sensitivity, nucleic acid amplification tests (NAATs) have been widely applied for rapid diagnosis of TB, among which the Xpert MTB/RIF assay, endorsed by the World Health Organization (WHO) in 2010, has been a significant advancement offering rapid detection of *Mycobacterium tuberculosis* and rifampicin resistance in less than 2 h. These techniques are based on the amplification of unique mycobacterial target sequences. Several appropriate genes, such as *16SrRNA*, *IS6110*, *IS1081*, *HSP65*, and *rpoB*, have been utilized for these molecular methods. However, most of the molecular assays that are currently in use only target one gene, which can lead to the misdiagnosis of tuberculosis (Comín et al. 2022; Huang et al. 2022; Pang et al. 2017; Jin et al. 2023; Chin et al. 2020). Meanwhile, due to point mutations in primer binding regions, low bacterial load, sample inhibitors, and assay limitations, false-negative results may occur, resulting in a missed diagnosis of tuberculosis (Phyu et al. 2018; Moradiya et al. 2020).

Capillary electrophoresis (CE) can separate charged macromolecules, such as DNA, which separates molecules based on their differential migration in an electric field. CE can separate DNA fragments of up to 1000 nucleotides with single-nucleotide resolution. It is a robust analytical technique with several potential advantages, such as short analysis time, low sample volume requirements (nanoliter or less), and high efficiency. For the most part, it has multianalyte capability, which allows it to assay multiple targets simultaneously. Furthermore, with flexible applications of parallel operation, CE has the potential for high-throughput analysis.

We therefore developed a CE-based multiplex molecular detection assay capable of detecting three MTBC genes: *IS6110*, *rpoB*, and *HSP65*. The selection of target genes was based on their established roles in MTBC identification and diagnostic utility. *IS6110* is a highly specific and multi-copy insertion sequence within the MTBC, widely used for molecular detection. *rpoB* encodes the β-subunit of RNA polymerase and is associated with rifampicin resistance; its inclusion allows for potential simultaneous detection of drug resistance. *HSP65* is a conserved heat shock protein gene frequently used for mycobacterial species differentiation. We then evaluated this method’s analytical performance and conducted clinical validation. By targeting these three genes simultaneously, we aim at enhancing diagnostic robustness, particularly in cases where one target may be absent or mutated.

## Materials and methods

### Primer design

Three pairs of oligonucleotides were required to detect the target genes of MTBC. Briefly, they were designed as follows: first, gene sequences were retrieved from NCBI for the following mycobacterium: *M. tuberculosis* (ATCC 27294), *M. marinum* (ATCC 927), *M. gordonae* (ATCC 14470), *M. scrofulaceum* (ATCC 19981), *M. terrae* (ATCC 15755), *M. fortuitum *subsp.* fortuitum* (ATCC 6841), *M. asiaticum* (ATCC 25276). Second, comparative analysis was performed through sequence alignment using Clustal Omega to obtain the conserved regions of the three target genes. Third, Primer Premier 5 software was used to design the primers according to the conserved regions. Then, the specificity of these forward primers was tested in silico using the BLAST tools from NCBI. To discriminate the three targets, these three forward primers were labelled with fluorophores, 6-FAM and TAMRA, respectively, at the 5′-end to be subsequently detectable by electrophoresis. Additionally, cross-reaction of these reverse primers should be avoided.

### DNA extraction

The Chelex-100 method was modified for DNA extraction. Specifically, two loops of freshly grown mycobacterial cultures from L-J medium were added to 300 μl of water containing Chelex resin. To improve the efficiency of DNA extraction, 10 μl proteinase K (10 mg/ml) was added to the DNA extraction process, considering the high lipid content in the MTBC membrane. Incubate at 56 °C for 20 min and then boil at 100 °C for 5 min to release DNA. Following this, the sample was centrifuged to pellet cellular debris and Chelex resin, and the supernatant was used as the DNA template. Then, DNA concentration and purity were determined by spectrophotometer (NanoDrop One, Thermo Scientific, USA).

### Polymerase chain reaction

In brief, the PCR reaction was carried out in a 50-μL mixture each containing 1.0 μL dNTP (10 mmol/L) (Invitrogen, USA), 2.0 μL AmpliTaq Gold™ DNA polymerase (5U/μL) (ABI, USA), 5.0 μL PCR Buffer(without Mg^2+^) (ABI, USA), 8.0 μL MgCl_2_(25 mmol/L) (ABI, USA), 26.0 μL amplification-grade water (Promega, USA), 0.5 μL each primer, and 5.0 μL DNA template. The reaction was performed using an automated thermal cycler (Verity, ABI, USA). The following PCR protocol was used: predenaturation of 94 ℃ for 5 min, 35 cycles of 94 ℃ for 30 s, 55 ℃ for 1 min, and 72 ℃ for 1 min, then a final extension step of 72 ℃ for 1 min.

### Capillary electrophoresis

The 3130 genetic analyzer (ABI, USA) was utilized. The procedures were briefly as follows: first, a 9.0-μL mixture of LIZ 500 (Promega, USA) and HiDi buffer (Promega, USA) in a 1:130 ratio was added to a 96-well plate. A 1.0-μL multiplex PCR product was then pipetted into each well, followed by a pre-denaturation process with heating the plate to 99 ℃ for 3 min. Then, capillary electrophoresis was performed according to the manufacturer’s instructions.

### Whole-genome and Sanger sequencing

Genomic DNA from MTBC isolates with discordant or irregular molecular assay results was sent to Majorbio (Shanghai, China) for whole-genome sequencing. To increase the accuracy and integrity of genome assembly, we employed a dual-platform strategy using both next-generation sequencing (Illumina) for high-depth short-read data and third-generation sequencing (PacBio) for long-read data. The raw Illumina reads were first quality-checked using FastQC and then filtered and trimmed. For long-read data, quality control was performed using the SMRT Analysis suite. Assembly was conducted using hybrid assembly approaches with SOAPdenovo and unicycler, which allowed us to leverage the strengths of both sequencing technologies. The assembled genomes were annotated using Prokka, and sequence alignments were performed with BLAST. For phylogenetic analysis, a set of 31 housekeeping genes was extracted from the assemblies, and the evolutionary relationships were constructed using MEGA 6.0 via the neighbor-joining method.

### Analytical evaluation

The analytical sensitivities of the multiplex assay were evaluated by determining the limit of detection (LOD). The LOD was measured using spiked samples with ATCC 27294. Twenty replicates were evaluated at five concentrations around LOD, and the LOD was determined using probit analysis. Then, studies were performed to determine the analytical specificity. Seventy-six (76) microorganisms (Table 1), which represent common respiratory pathogens, were tested at the following concentrations: DNA at 1 × 10^7^ copies/mL for bacteria and fungi, nucleic acid at 2 × 10^9^ copies/mL for viruses, DNA at 1 × 10^5^ copies/mL for nontuberculous mycobacteria, and a concentration of 10^6^ elementary bodies (EB) per mL for Chlamydia. All specificity and sensitivity assays were tested in duplicate.

**Table 1:** Microorganisms tested for analytical evaluation

Microorganisms	Origin	Microorganisms	Origin
*Mycobacterium gastri*	ATCC15754	*Haemophilus influenzae*	Clinical isolates
*Mycobacterium terrae*	ATCC15755	*Haemophilus parainfluenzae*	Clinical isolates
*Mycobacterium xenopi*	ATCC19250	*Klebsiella oxytoca*	Clinical isolates
*Mycobacterium smegmatis*	ATCC19420	*Klebsiella pnenmoniae*	Clinical isolates
*Mycobacterium ulcerans*	ATCC19423	*Legionella pneumophila*	Clinical isolates
*Mycobacterium thermoresistibile*	ATCC19527	*Micrococcus luteus*	Clinical isolates
*Mycobacterium abscessus*	ATCC19977	*Morganella morganii*	Clinical isolates
*Mycobacterium scrofulaceum*	ATCC19981	*Mycobacterium fortuitum*	Clinical isolates
*Mycobacterium simiae*	ATCC25275	*Mycobacterium gordonae*	Clinical isolates
*Escherichia coli*	ATCC25922	*Mycobacterium intracellulare*	Clinical isolates
*Staphylococcus aureus*	ATCC25923	*Mycobacterium kansassi*	Clinical isolates
*Pseudomonas aeruginosa*	ATCC27853	*Neisseria meningitidis*	Clinical isolates
*Mycobacterium haemophilum*	ATCC29548	*Nocardia asteroides*	Clinical isolates
*Mycobacterium malmoense*	ATCC29571	*Nocardia brasiliensis*	Clinical isolates
*Shigella flexneri*	ATCC29903	*Nocardia farcinica*	Clinical isolates
*Mycobacterium chelonae*	ATCC35752	*Proteus mirabilis*	Clinical isolates
*Mycobacterium szulgai*	ATCC35799	*Proteus vulgaris*	Clinical isolates
*Mycobacterium celatum*	ATCC51131	*Pseudomonas aeruginosa*	Clinical isolates
*Mycobacterium genavense*	ATCC51234	*Pseudomonas fluorescens*	Clinical isolates
*Mycobacterium marinum*	ATCC927	*Pseudomonas putida*	Clinical isolates
*Mycobacterium colombiense*	CIP108962	*Serratia marcescens*	Clinical isolates
*Mycobacterium paraintracellulare*	JCM30622	*Shigella boydii*	Clinical isolates
*Mycobacterium Massiliense*	CIP108297	*Staphylococcus capitis*	Clinical isolates
*Mycobacterium bolletii*	DSM45149	*Staphylococcus epidermidis*	Clinical isolates
*Mycobacterium avium*	Clinical isolates	*Staphylococcus haemolyticus*	Clinical isolates
*Acinetobacter baumannii*	Clinical isolates	*Staphylococcus saprophyticus*	Clinical isolates
*Acinetobacter calcoaceticus*	Clinical isolates	*Stenotrophomonas maltophilia*	Clinical isolates
*Acinetobacter haemolyticus*	Clinical isolates	*Streptococcus agalactiae*	Clinical isolates
*Acinetobacter junii*	Clinical isolates	*Streptococcus midis*	Clinical isolates
*Alcaligenes faecalis*	Clinical isolates	*Streptococcus salivarius*	Clinical isolates
*Branhamella catarhalis*	Clinical isolates	*Adenovirus*	NIFDC 370057-202001
*Candida albicans*	Clinical isolates	*Coronavirus*	NIFDC 370057-202001
*Citrobacter freundii*	Clinical isolates	*Human metapneumovirus*	NIFDC 370057-202001
*Corynebacterium diphtheriae*	Clinical isolates	*Influenza B Virus*	NIFDC 370057-202001
*Enterobacter aerogenes*	Clinical isolates	*Parainfluenza virus*	NIFDC 370057-202001
*Enterobacter cloacae*	Clinical isolates	*Respiratory syncytial virus*	NIFDC 370057-202001
*Enterococcus faecalis*	Clinical isolates	*Rhinovirus*	NIFDC 370057-202001
*Enterococcus faecium*	Clinical isolates	*Rubella virus*	NIFDC 370057-202001

ATCC: American Type Culture Collection; CIP: Collection of Institut Pasteur, France; JCM: Japan Collection of Microorganisms; DSM: Deutsche Sammlung von Mikroorganismen und Zellkulturen, German; NIFDC: National Institutes for Food and Drug Control, China

### Clinical performance

A prospective cohort study was conducted to evaluate the performance characteristics of the multiplex assay. Inclusion criteria: Subjects 18 years or older were eligible for the study if they were suspected of pulmonary tuberculosis (based on symptoms such as persistent cough, fever, weight loss, night sweats, and/or abnormal chest radiography findings) in Fujian Provincial Hospital, China, and on no TB treatment or with less than 3 days of TB treatment. Exclusion criteria: insufficient sputum volume. Then, we estimated that a sample size of 1100 patients is needed to obtain a between-group difference with more than 99% power. Meanwhile, this is an adequate sample size for evaluating the sensitivity and specificity according to the CLSI guideline. Testing should continue until results from at least 50 positive specimens, as a minimum guideline, are obtained with both the test and comparative method. The Institutional Ethics Committee of Fujian Provincial Hospital approved the study.

Sputum specimens collected from subjects were tested using AFB smear, culture, and Xpert MTB/RIF. Also, a single leftover sputum sample was tested using the multiplex assay. All the participants were at least independently diagnosed by two doctors based on clinical and laboratory findings. If the diagnosis is different, another doctor is required. Then, the sensitivity, specificity, negative predictive value (NPV), and positive predictive value (PPV) of the four methods were calculated, respectively.

Discordant results for MTBC culture positive and molecular assay negative, irrespective of Xpert MTB/RIF or multiplex assay, were further evaluated using bi-directional sequencing of the corresponding region of the genome. If needed, WGS was used for further validation.

### Data analysis

Statistical analyses were performed using SPSS Statistics 24, including the sensitivity, specificity, NPV, and PPV of the four methods adopted in this study, and their corresponding 95% confidence interval (CI). Probit analysis was used to determine the LOD of the multiplex assay. The LOD was defined as the lowest DNA concentration detected in 95% of 20 replicates for every target gene.

## Result

### CE-based multiplex assay

Specific primers (Table 2) targeted *IS6110*, *rpoB*, and *HSP65* were designed based on their conserved regions, respectively. Then, the specificity of these forward primers was examined in silico. Compared with the core nucleotide BLAST database, which consists of 108,970,392 sequences, the number of hits for the forward primer targeted *IS6110* is 101, 99 for MTBC, and 2 for *Mycobacterium avium* complex. For *rpoB*, the number of hits is 109, which includes 2 for *Pontimonas *sp. and 107 for MTBC. The number of hits for the forward primer targeted *HSP65* is 101, which is all for MTBC. Moreover, the identity of all these hits above is 100%.

**Table 2:** Oligonucleotides used for amplification by PCR

Gene	Oligonucleotide	Sequence	Fluorophores	Size
*IS6110*	*IS6110*-F	5’-TACGGTGCCCGCAAAGTG-3’	5'-TAMRA	271bp
	*IS6110*-R	5’-AGGCGTCGGTGACAAAGG-3’	/	
*rpoB*	*rpoB*-F	5'-CCAATTCATGGACCAGAACAA-3'	5'−6-FAM	270 bp
	*rpoB*-R	5'-TACACGATCTCGTCGCTAACC-3'	/	
*HSP65*	*HSP65*-F	5'-AGCGATTTCGGCGGGTGA-3'	5'−6-FAM	346 bp
	*HSP65*-R	5'-TCTTGTTGACGACCAGGGTG-3'	/	

**Fig. 1:**
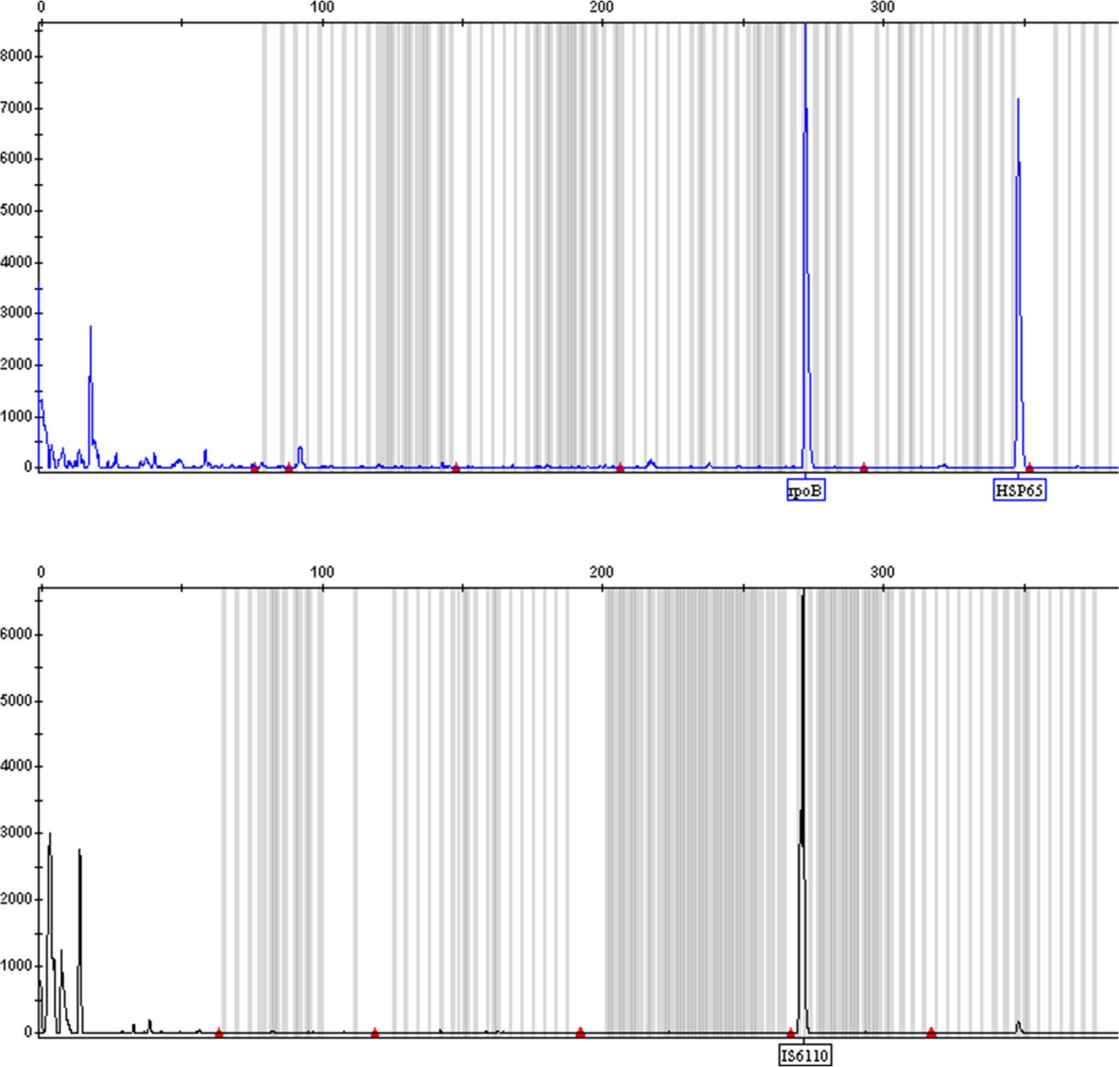
The result of *Mycobacterium tuberculosis* H37Rv (ATCC 27294) using multiplex assay

The result of *Mycobacterium tuberculosis* H37Rv (ATCC 27294) using this multiplex assay is in line with expectations (Fig. 1). After targeted DNA sequencing and alignment with reference sequence, the identity is 99.3% (269/271), 99.3% (268/270), and 98.8% (342/346) for *IS6110*, *rpoB*, and *HSP65*, respectively (Fig. S1).

### Analytical evaluation

Three target genes were all successfully detected by the multiplex assay with an LOD ranging from 10 to 20 copies/mL (Table 3). Irrespective of the type of microorganisms, genomic DNA extracts from 76 respiratory pathogens were not detected by the multiplex method. So, the analytical specificity of the multiplex assay is 100% at the given concentration regarding bacteria, fungi, viruses, Chlamydia, and nontuberculous mycobacteria.

**Table 3:** Detection limit of Multiplex assay for three target genes

Gene	Multiplex assay			
	Limit of detection (copies/mL)^a^	Number of replicates	Number detected	% detected
*IS6110*	10	20	20	100
*rpoB*	20	20	19	95
*HSP65*	20	20	20	100

^a^ The limit of detection (LOD) was defined as the lowest DNA concentration that was detected in 95% of 20 replicates

### Clinical performance

**Fig. 2:**
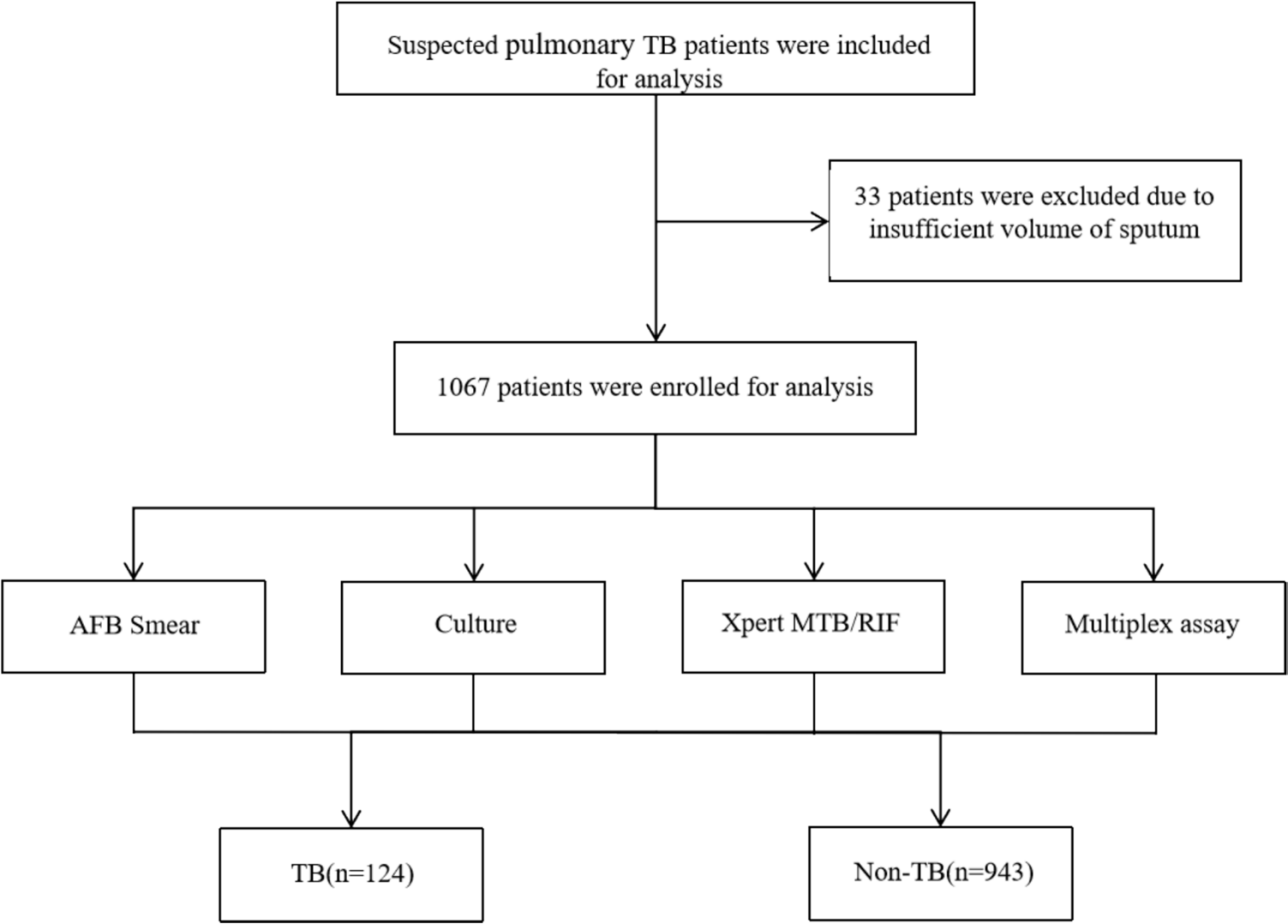
The flowchart of patients included in this study

A total of 1100 patients were enrolled between January 2023 and December 2023, excluding 33 patients due to insufficient sputum volume. The final enrollment of 1067 patients was performed by AFB smear, culture, Xpert MTB/RIF, and the multiplex assay. Figure 2 shows the classification of patients included in this study. In total, 124 patients were diagnosed with pulmonary tuberculosis. The overall positive rates of AFB smear, culture, Xpert MTB/RIF, and the multiplex assay, not taking the diagnosis into account, were 4.9%, 11.8%, 10.0%, and 9.4%, respectively (Table 4). However, for the TB patients, the positive rates were 25.8%, 75.0%, 79.8%, and 77.4%, respectively.

**Table 4:** Performance of different methods to diagnose TB in the cohort

Detection Methods	Test Results	Rate	Diagnosis		Sensitivity	Specificity	PPV	NPV
			TB	Non-TB	CI	CI	CI	CI
AFB smear	Positive	4.9%	32(25.8%)	21(2.2%)	25.8%	97.7%	60.4%	90.9%
	Negative	95.1%	92(74.2%)	922(97.7%)	23.2%−28.4%	96.9%−98.7%	57.4%−63.3%	89.2%−92.7%
Culture	Positive	11.8%	93(75.0%)	32(3.3%)	75.0%	96.7%	74.4%	96.7%
	Negative	88.2%	31(25.0%)	911(96.7%)	72.4%−77.6%	95.5%−97.7%	71.8%−77.0%	95.6%−97.8%
Xpert MTB/RIF	Positive	10.0%	99(79.8%)	8(0.8%)	79.8%	99.2%	92.5%	97.4%
	Negative	90.0%	25(20.2%)	935(99.2%)	77.4%−82.3%	98.6%−99.7%	91.0%−94.1%	96.4%−98.4%
Multiplex assay	Positive	9.4%	96(77.4%)	4(0.4%)	77.4%	99.6%	96.0%	97.1%
	Negative	90.6%	28(22.6%)	939(99.6%)	74.9%−79.9%	99.2%−100%	94.8%−97.2%	96.1%−98.1%

PPV, Positive Predictive Value. NPV, Negative Predictive Value. CI, 95% upper and lower confidence interval

Comparisons of the four methods for the MTBC are given in Table 4. In total, 93 patients tested positive for multiplex assay, all of which were culture-positive. However, 31 patients who were not finally diagnosed with TB were culture-positive, all of which were multiplex assay–negative. Conversely, other 4 non-TB patients, who were diagnosed as obsolete pulmonary tuberculosis, were Xpert MTB/RIF positive and multiplex assay–positive, none of which was culture-negative. The overall sensitivity of AFB smear for all 1067 patients was 25.8% (CI 23.2%–28.4%), specificity 97.7% (CI 96.9%–98.7%), PPV 60.4% (CI 57.4%–63.3%), and NPV 90.9% (CI 89.2%–92.7%). The corresponding values for culture were 75.0% (CI 72.4%–77.6%), 96.7% (CI 95.5%–97.7%), 74.4% (CI 71.8%–77.0%), and 96.7% (CI 95.6%–97.8%). Meanwhile, the multiplex assay showed 77.4% (CI 74.9%–79.9%) sensitivity, 99.6% (CI 99.2%–100%) specificity, 96.0% (CI 94.8%–97.2%) PPV, and 97.1% (CI 96.1%–98.1%) NPV.

### Identification of *IS6110*-like element in MTBC

**Fig. 3:**
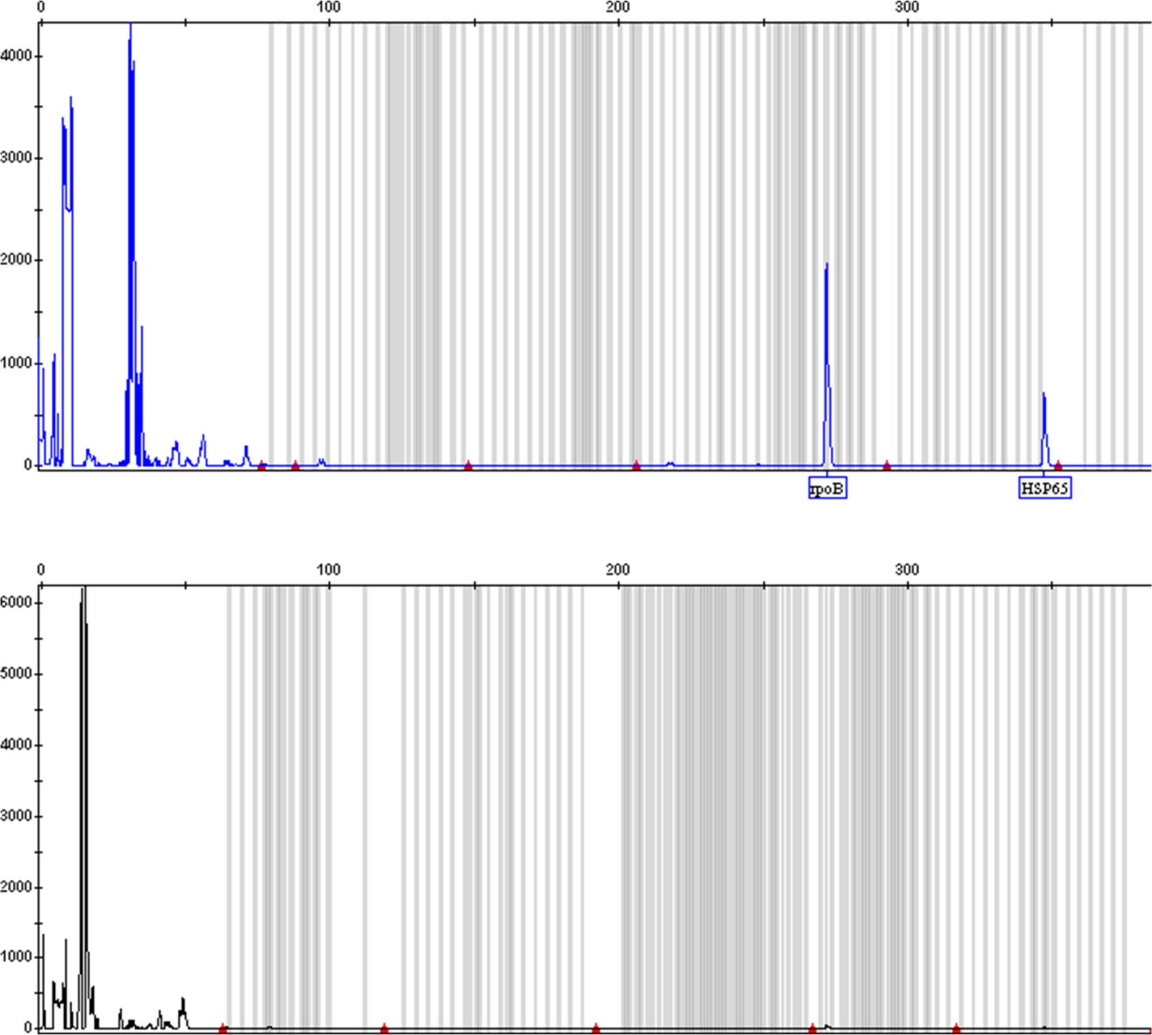
Example of the result of TB patients (*IS6110* deletion) using multiplex assay

**Fig. 4:**
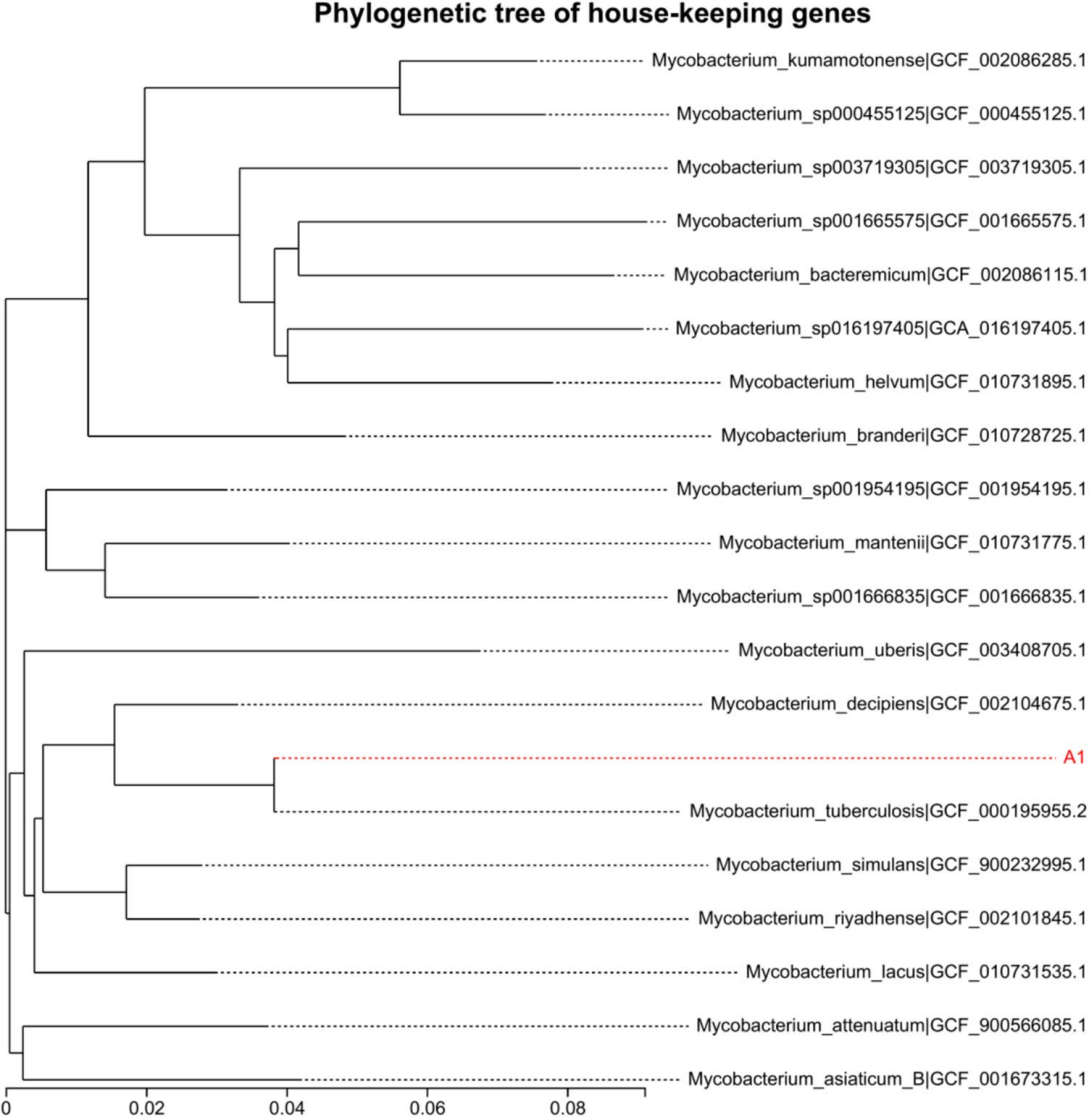
Phylogenetic tree of A1 isolate. The phylogenetic analysis was conducted based on *dnaG, frr, infC, nusA, pgk, pyrG, rplA, rplB,rplC, rplD, rplE, rplF, rplK, rplL, rplM, rplN, rplP, rplS, rplT, rpmA, rpoB, rpsB, rpsC, rpsE, rpsI, rpsJ, rpsK, rpsM, rpsS, smpB*, and *tsf* 31 housekeeping genes, using MEGA 6.0 by neighbor-joining method

Among the 93 patients who were culture, Xpert MTB/RIF and multiplex assay triple positive, we could not detect *IS6110* element in 6 TB patients using the multiplex assay (Fig. 3). Due to the possibility of misdiagnosis, a more careful analysis of the 6 isolates was carried out using WGS. The evolutionary relationship of the 6 isolates, based on *dnaG*, *frr*, *infC*, *nusA*, *pgk*, *pyrG*, *rplA*, *rplB*, *rplC*, *rplD*, *rplE*, *rplF*, *rplK*, *rplL*, *rplM*, *rplN*, *rplP*, *rplS*, *rplT*, *rpmA*, *rpoB*, *rpsB*, *rpsC*, *rpsE*, *rpsI*, *rpsJ*, *rpsK*, *rpsM*, *rpsS*, *smpB*, and *tsf* 31 housekeeping genes, is displayed in Fig. 4 (take A1 isolate as an example). Phylogenetic analysis revealed that the 6 isolates most likely belonged to *Mycobacterium tuberculosis*.

**Fig. 5:**
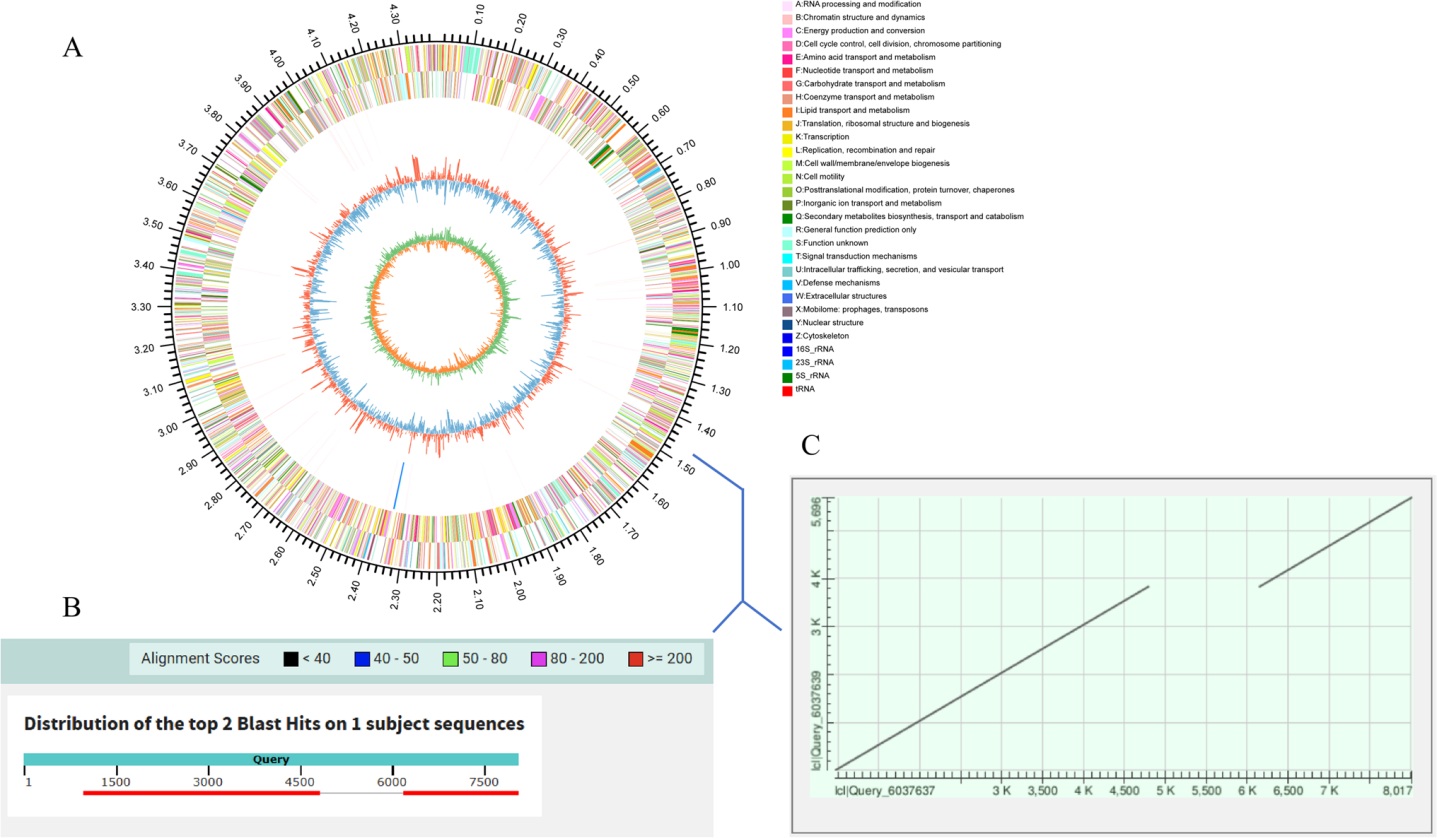
The location of *IS6110* deletion. **A** Circos plot of the genome of A1 strain. **B** Alignment of sequence in the location of *IS6110* deletion with *Mycobacterium tuberculosis* H37Rv reference sequence. **C** Dot plot of the alignment

Furthermore, the whole genomes were obtained after genome assembly (Fig. 5A). As expected, we could not find the oligonucleotide sequence designed for *IS6110* in these genomes. Neither did we find the reverse complementary sequence. Since *IS6110* was multicopy and belonged to mobile genetic elements, we attempted to position one deletion in these genomes. Then, sequence alignment with *Mycobacterium tuberculosis* H37Rv reference sequence (accession ID NC_000962) was carried out using BLAST tools. Finally, we positioned one deletion at location: 1463537 (Fig. 5B, C). In comparison with the reference sequence, we found an approximate 1500-bp deletion did exist in the *IS6110* corresponding region.

## Discussion

About a quarter of the population is globally estimated to have been infected with MTBC (Cohen et al. 2019). Rapid and accurate detection of MTBC is of great significance in controlling the TB spread, a devastating infectious disease (Naidoo et al. 2022). Nowadays, various commercially available NAATs kits are adopted in clinical laboratories worldwide (Mousavi-Sagharchi et al. 2024). Also, various novel NAATs methods are developed for MTBC detection (Wang et al. 2024; Batuer et al. 2024; Svensson et al. 2021; Yee et al. 2020; Huang et al. 2022; Homann et al. 2021; Shan et al. 2022). However, most of these molecular kits only target one gene. This can lead to the misdiagnosis and missed diagnosis of TB. Misdiagnosis of TB can bring about substantial negative consequences (Houben RMGJ et al. 2019). Firstly, a treatment course that lasts at least 6 months will be falsely recommended. Also, patients will incur substantial costs for clinical services and nonclinical expenses, like transportation, food, childcare, and lost wages (Laurence et al. 2015). Meanwhile, missed diagnosis of TB can result in the delay of treatment for patients, which can do harm to TB prevention (Medrano et al. 2014). Here, we propose a novel, rapid, and multi-target method that can detect three genes of MTBC simultaneously, aiming to provide a more comprehensive method for MTBC to reduce the misdiagnosis and missed diagnosis of TB.

This is the first study that developed a multiplex assay to detect MTBC based on the CE platform. To ensure the specificity of this method, a two-step strategy was employed to design PCR primers. First, the conserved regions of the MTBC were identified through sequence alignment with several common NTM. Then, the BLAST tools from NCBI were utilized to test the specificity of these primers, which were designed based on the conserved regions. Specifically, compared with the core nucleotide BLAST database, it showed probable cross-reactivity with *Pontimonas *sp. for *rpoB* and *Mycobacterium avium* complex for *IS6110*, proving no cross-reaction occurred by follow-up experiments. Also, here we report the performance of this method in a cohort, with 1067 patients finally enrolled, compared with AFB smear, culture, and Xpert MTB/RIF. The multiplex assay exhibited a sensitivity of 77.4% vs. 25.8% of AFB smear, 75.0% of culture, and 79.8% of Xpert MTB/RIF. The increment in sensitivity of the multiplex assay over AFB smear was significant, whereas the increment in sensitivity of the multiplex assay over culture was marginal. Also, we observed a low increment in specificity and NPV of the multiplex assay over AFB smear, culture, and Xpert MTB/RIF (specificity 99.6% vs. 97.7%, 96.7%, and 99.2%, respectively, NPV 97.1% vs. 90.9%, 96.7%, and 97.4%, respectively). However, regarding PPV, the corresponding value of the multiplex assay was 96.0% vs. 60.4%, 74.4%, and 92.5% for AFB smear, culture, and Xpert MTB/RIF, respectively. Xpert MTB/RIF is acknowledged to be an excellent method to detect MTBC (WHO 2020). In this context, it is worth noting that, with more target genes to be detected simultaneously, the multiplex assay performed at least as well as Xpert MTB/RIF.

Compared to the Xpert MTB/RIF assay, which is a fully integrated, cartridge-based system suitable for point-of-care settings, our CE-based approach is more suitable for centralized laboratories with existing capillary electrophoresis infrastructure. In terms of cost per test, our method may be more economical in high-throughput settings, though it requires an initial investment in CE equipment. A detailed comparison of key characteristics between our assay and the Xpert MTB/RIF system is provided in Table S1. The primary advantage of our assay lies in its multi-target design, which enhances diagnostic accuracy, particularly in regions with circulating *IS6110*-deficient strains.

During the clinical evaluation of the multiplex assay, we discovered 6 strains of MTBC with suspected *IS6110* deletion. First, we adopted Sanger sequencing for *IS6110*, *rpoB*, and *HSP65*, three genes, to identify these strains. Regarding *rpoB* and *HSP65*, the results were in line with the reference sequence of *Mycobacterium tuberculosis* (data not shown). Nevertheless, we could only obtain the *IS6110* sequence result if we designed these primers. Then, the whole genomes were obtained using WGS based on the Illumina and Pacbio platforms. According to the phylogenetic analysis based on 31 housekeeping genes, these 6 strains belonged to *Mycobacterium tuberculosis*. Moreover, after the genome assembly, an approximate 1500-bp deletion did exist in the *IS6110* corresponding region. Owing to this finding, we could also recognize that the long gap was why we failed to get the results by Sanger sequencing. Certainly, in this regard, it would lead to a false negative result for any commercial PCR kits targeted at *IS6110*. Although we just discovered 6 *IS6110* deletion strains (4.8%, 6/124) in our study, it was reported that the corresponding frequency was about 8% and 11% in Viet Nam and India, respectively (Chauhan et al. 2007). This indicates the necessity of the multiplex assay we developed here to reduce the missed diagnosis of TB.

The study has limitations. First, clinical validation of this multiplex assay was performed on just one site, which may lead to bias. Second, considering sputum volume, we select Xpert MTB/RIF targeted *rpoB* as a comparison in the cohort, endorsed by WHO (WHO 2020). So, in this regard, another two molecular assays, targeted *IS6110* and *HSP65*, respectively, were needed. Third, although 76 microorganisms, which represent common respiratory pathogens, were selected for analytical specificity, we did not take *Pontimonas *sp., which showed 100% identity aligned with the oligonucleotide designed for *rpoB*, into evaluation, since the corresponding strain was not available in our library.

In conclusion, the multiplex assay simultaneously provides one-run results for *IS6110*, *rpoB*, and *HSP65*, maintaining adequate sensitivity, specificity, PPV, and NPV for TB diagnosis. We present a more comprehensive method to detect MTBC. Although this is only a small fraction of the overall endeavor for accurate diagnosis, the multiplex assay has the potential to reduce misdiagnosis and missed diagnosis of TB. To achieve the global end TB goals, the multiplex assay, a comprehensive alternative to methods currently used, deserves further field evaluation.

## Supplementary Information

Below is the link to the electronic supplementary material.

ESM 1 (DOCX 13.5 KB)

## Data Availability

Not applicable.
