# Supplementary material for: A multiplex assay based on capillary electrophoresis to detect Mycobacterium tuberculosis complex: development and clinical validation
Source: Appl Microbiol Biotechnol. 2026 Feb 2;110(1):54. doi: 10.1007/s00253-025-13701-0 (PMC12868050; doi:10.1007/s00253-025-13701-0)
Supplement: Supplementary file 1 — (DOCX 13.5 KB) [file 253_2025_13701_MOESM1_ESM.docx]

**Supplementary information**

**Fig. S1.** The alignment with reference sequence. (A) *IS6110*. (B) *rpoB*. (C) *HSP65*.

**TABLE S1**

Comparative summary of the Multiplex assay and Xpert MTB/RIF assay

| Feature | Multiplex Assay | Xpert MTB/RIF Assay |
| --- | --- | --- |
| Target genes | *IS6110*, *rpoB*, *HSP65* (three targets) | *rpoB* (single target) |
| Detection principle | Capillary electrophoresis (CE) coupled with multiplex PCR | Integrated real-time PCR in a cartridge-based system |
| Primary diagnostic advantage | Multi-target detection reduces false negatives due to target deletion/mutation (e.g. *IS6110*-deficient strains) | Rapid, simple, and suitable for point-of-care use; detects MTB and rifampicin resistance simultaneously |
| Equipment requirement | CE instrument (e.g.ABI 3130) and thermal cycler | Dedicated GeneXpert instrument |
| Time to result | ~3 hours (including DNA extraction, PCR, and CE analysis) | <2 hours |
| Cost per test  (excluding equipment) | Lower in high-throughput settings (reagent costs only) | Higher  (includes disposable cartridge cost) |
| Simultaneous drug resistance detection | No (but *rpoB* target provides a basis for future resistance assay development) | Yes (rifampicin resistance via  *rpoB* mutations) |
| Sample volume required | 1 µL of PCR product for CE | 1–2 mL of sputum sample for processing |
| Throughput capability | High  (parallel analysis of 96 samples per run) | Moderate (1–4 samples per module) |
| Hands-on time | Moderate (requires PCR setup and CE plate loading) | Minimal (fully automated processing after sample loading) |
| Ideal setting | Centralized laboratories with existing CE infrastructure | Decentralized settings, clinics, point-of-care locations |
